# Effectiveness and safety of music-supported therapy on mood in post-stroke rehabilitation patients: A protocol for systematic review and meta-analysis

**DOI:** 10.1097/MD.0000000000025077

**Published:** 2021-03-26

**Authors:** Jie Ding, Yuanyi Xiao, Fuqiang Yuan, Zhaona Luo, Jinyu Hu

**Affiliations:** a College of Music, Jiangxi Science and Technology Normal University; b College of Acupuncture-Moxibustion and Tuina, Jiangxi University of Traditional Chinese Medicine; c The Affiliated Hospital of Jiangxi University of Traditional Chinese Medicine, Nanchang, China.

**Keywords:** mood disorders, music-supported therapy, protocol, stroke, systematic review

## Abstract

**Background:**

Music-supported therapy has been widely used clinically to relieve post-stroke rehabilitation. However, the efficacy of Music-supported therapy in the treatment of Mood in post-stroke rehabilitation Patients is uncertain. The purpose of this study is to determine the effectiveness and safety of Music-supported therapy in the treatment of Mood in post-stroke rehabilitation Patients.

**Methods:**

Search PubMed, Cochrane Library, Embase, China National Knowledge Infrastructure Database, Wanfang Database, China Science and Technology Journal Database, China Biomedical Literature Database, and search related randomized controlled trials. Two reviewers will independently select studies, collect data, and evaluate methodological quality through the Cochrane Deviation Risk Tool. Revman V.5.3 will be used for meta-analysis.

**Results:**

This study will evaluate the current status of Music-supported therapy treatment for mood in post-stroke rehabilitation Patients, aiming to illustrate the effectiveness and safety of Music-supported therapy treatment.

**Conclusion:**

This study will provide a basis for judging whether Music-supported therapy is effective in treating mood in post-stroke rehabilitation Patients.

**INPLASY registration number:**

INPLASY202120011.

## 1 Introduction

Stroke is one of the main diseases that seriously damage human health. It has the characteristics of high morbidity, high disability and high mortality.^[1]^ The main clinical manifestations of post-stroke patients are long-term physical and psychological problems, including movement and function, balance, pain, sensation, perception, cognition, attention, memory, and emotional problems.^[2]^ Among them, motor dysfunction, anxiety and depression are the most important factors that hinder patients from participating in daily activities after stroke and restrict their occupational rehabilitation.^[3,4]^ According to epidemiological studies Post-stroke depression is presented in 32.9% to 35.9% of stroke patients, which is significantly higher than the prevalence of depression in the general population (10%).^[5]^ Rehabilitation after stroke has caused many social problems, including patients’psychological depression and increasing the country's overall medical expenses.^[6]^

The etiology of stroke was divided by hemorrhage and infarction, In addition, other evidence suggests it is usually caused by many factors such as the obesity epidemic, diabetes, heart failure, and the overall lack of physical activity among the general population.^[7]^ At present, major goals of treatment are to reduce depressive symptoms, improve mood and quality of life, and reduce the risk of medical complications as well as the relapse of post-stroke depression.^[8]^ However, antidepressants are generally not indicated in mild forms because the balance of benefit and risk is not satisfactory in stroke patients.^[9]^

Music-supported therapy (MST) is a promising new treatment,^[10]^ and extensive research suggests that it could be useful because of its promotion of relaxation and both cognitive and motor improvement in stroke rehabilitation.^[11]^ There are many studies with music therapy on mood in post-stroke patients, although music therapy has been used in rehabilitation to stimulate brain functions involved in emotion, cognition, speech, and sensory perceptions.^[12,13]^

The focus of this study is the efficacy of Music-supported therapy on mood in post-stroke rehabilitation Patients. Therefore, we conducted this study to systematically evaluate the impact of Music-supported therapy on post-stroke rehabilitation Patients. It can provide a basis for the diagnosis and treatment of MST for post-stroke rehabilitation Patients.

## 2 Methods

### 2.1 Inclusion criteria for study selection

#### 2.1.1 Types of studies

All randomized controlled trials of Music-supported therapy for the post-stroke rehabilitation Patients will be included without language restriction. Non- randomized controlled trials, observational studies, cross-over studies, uncontrolled trials, animal trials, and reviews will be excluded.

#### 2.1.2 Types of participants

Inclusion criteria for study populations will be all patients with Anxiety and depression in post-stroke rehabilitation Patients. No restrictions will be applied in terms of gender, age, race, condition duration or intensity.

#### 2.1.3 Types of interventions

##### 2.1.3.1 Experimental interventions

The treatment group will only receive Music-supported therapy alone, without any restrictions on music material, type or treatment process.

##### 2.1.3.2 Control interventions

The control group will receive an internationally recognized therapy such as pharmacological therapies. Placebo, no treatment, and Sound wave will also be included. Studies that compare the effect of different types of music will be excluded.

#### 2.1.4 Types of outcome measures

##### 2.1.4.1 Primary outcomes

Mini mental status examination score and he Beck Depression Inventory and Beck Anxiety Inventory, Percentage of Clinical Efectiveness will be accepted as the primary outcomes.

##### 2.1.4.2 Additional outcomes

The safety assessment will be considered a secondary outcomes.

### 2.2 Search methods for the identification of studies

#### 2.2.1 Electronics searches

The following electronic databases will be searched: PubMed, Embase, the Cochrane Library, the China National Knowledge Infrastructure, Chinese Science and Technology Periodical Database, Wanfang Database, and Chinese Biomedical Literature Database. We will search the databases from the beginning to January 2021. Search terms consist of disease (post-stroke, stroke with Anxiety and depression, Cerebrovascular Accident, Cerebrovascular Apoplexy with Anxiety and depression) and intervention (Music Therapy, Acoustic Stimulation, music-supported therapy) and research types (randomized controlled trial, controlled clinical trial, random trials). The PubMed search strategy is shown in Table 1.

**Table 1:** Search strategy used in PubMed database.

Number	Search items
#1	randomized controlled trial [pt]
#2	controlled clinical trial [pt]
#3	randomized [tiab]
#4	clinical trials as topic [mesh: noexp]
#5	randomly [tiab]
#6	trial [ti]
#7	OR/ #1–#7
#8	animals [mh] NOT humans [mh]
#9	#7 NOT #8
#10	Stroke [Mesh]
#11	Cerebrovascular Accident [All Fields)
#12	Cerebrovascular Apoplexy [All Fields)
#13	Brain Vascular Accident [All Fields)
#14	Cerebrovascular Stroke [All Fields)
#15	Apoplexy[All Fields)
#16	Cerebral Stroke [All Fields)
#17	Acute Stroke [All Fields)
#18	Acute Cerebrovascular Accident
#19	OR/#10–#18
#20	Mood Disorders OR Affective Disorder OR Bipolar Disorders OR Manic-Depressive OR Manic-Depressive Psychosis OR Bipolar Mood Disorder OR Bipolar Depression OR Manic Depression ORManic DisorderOR anxiety OR depression[All Fields)
#21	#19 AND #20
#22	Music Therapy [Mesh]
#23	Acoustic Stimulation [All Fields)
#24	music-supported therapy [All Fields)
#25	OR/ #22–#24
#26	#9 AND #21 AND #25

#### 2.2.2 Search for other resources

We will also retrieve the relevant conference papers, and search for new trials related to music Therapy treatment on mood in post-stroke rehabilitation patients on the WHO International Clinical Trials Registration Platform and the Clinical Trials.gov.

### 2.3 Data collection and analysis

#### 2.3.1 Selection of studies

**Figure 1:**
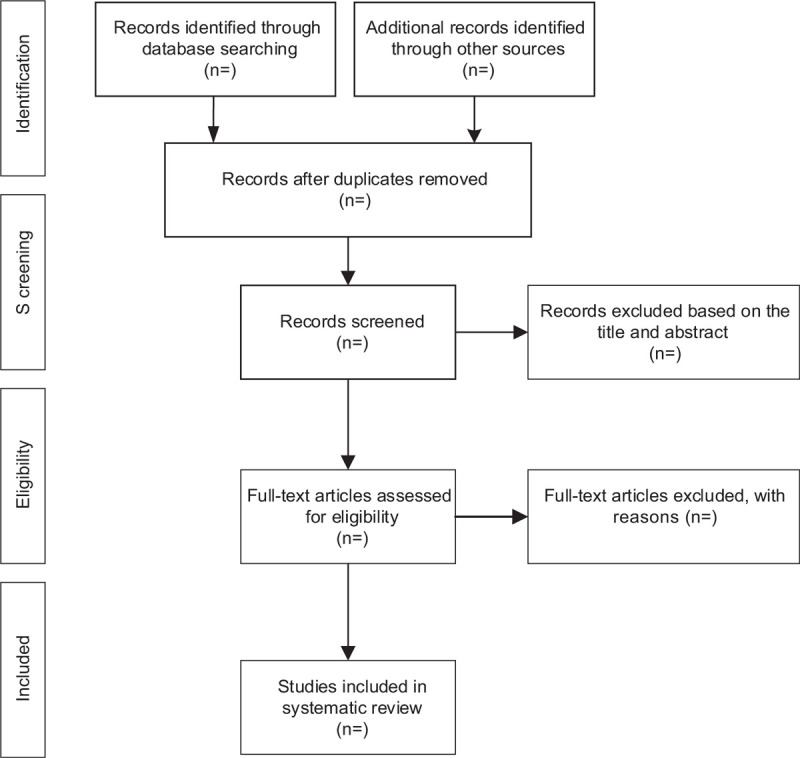
Flow diagram of study selection process.

We will import the retrieved literature into EndNote X7 software and delete the duplicate data. After that, two reviewers will independently scan the titles and abstracts. Unrelated literature will be deleted. If they cannot determine whether to include the study, they will obtain the full text of the article for judgment. Two reviewers will independently evaluate the eligibility of these articles based on inclusion and exclusion criteria. Any disagreements will be resolved through group discussions. The study selection procedure is shown in Figure 1.

#### 2.3.2 Data extraction and management

The data extraction for eligible studies will be completed independently by 2 authors, and any disagreement will be resolved through discussion with the third author. The extracted data will mainly include the first author, time of publication, patient characteristics, sample size, interventions, follow-up period, outcome measures and adverse events. If necessary, we will try to contact the author for the details by email.

### 2.4 Risk of bias assessment

Two independent authors will evaluate the risk of bias among the final included studies using the risk of bias assessment tool by the Cochrane Collaboration.^[14]^ The contents will include:

(1) random sequence generation;
(2) allocation concealment;
(3) blinding of participants and personnel;
(4) blinding of outcome assessment;
(5) incomplete outcome data;
(6) selective reporting; and
(7) other sources of bias.

Each study will be evaluated as High, Low, or Unclear risk of bias for each item. Discrepancies will be resolved through further discussion with the third author.

### 2.5 Quantitative data synthesis and statistical methods

#### 2.5.1 Quantitative data synthesis

We will conduct statistical analysis through RevMan 5.3 software. For Categorical data, we will calculate with the risk ratio and 95% confidence intervals. For continuous variables, mean difference will be included in the meta-analysis. If outcome variables are measured on different scales, results will be reported as standardized mean differences with 95% confidence interval.

#### 2.5.2 Assessment of heterogeneity

We will use chi-square test and *I*^*2*^ test to evaluate the statistical heterogeneity. When *P* > .10 and *I*^*2*^≤50%, the research results will not be considered heterogeneous; otherwise, it will be considered as heterogeneous.

#### 2.5.3 Assessment of reporting biases

When more than 10 studies are included, funnel plot will be generated to detect the reporting bias. In addition, we will use the Egger test to check the asymmetry of funnel plot.

#### 2.5.4 Subgroup analysis

If the included studies have significant heterogeneity, we will perform subgroup analysis based on different control groups.

#### 2.5.5 Sensitivity analysis

When sufficient studies are available, sensitivity analysis will be used to assess the robustness of the meta-analysis based on methodological quality, sample size and missing data.

#### 2.5.6 Grading the quality of evidence

We will assesse the quality of evidence by the Grading of Recommendations Assessment, Development and Evaluation and rate it into high, moderate, low or very low 4 levels.^[15,16]^

## 3 Discussion

MST has been widely used in the treatment of post-stroke rehabilitation Patients, and the Stroke Rehabilitation Guidelines also recommend music neurorehabilitation as a supportive treatment approach. Neurorehabilitation with music is supported by studies showing how music facilitates neural connections and neuronal reorganization of the sensorimotor cortex.^[17]^ A high-quality randomized controlled trial clinical study shows that Music Therapy can effectively improve motor functions (in particular gait functions and upper limbs motor skills)^[18]^ or language functions.^[19]^ In addition, related basic experiments have proved that music induced adjustment of respiratory rhythm, relaxation of muscular stiffness,^[20]^ decrease of heart rate and blood pressure by formation of a comfortable atmosphere, and alleviation of tension by increased alpha waves in the brain,^[21]^ but the clinical efficacy of MST has not been scientifically and systematically evaluated. This study aims to evaluate the clinical efficacy and safety of MST in the treatment of post-stroke rehabilitation Patients. The conclusions of this study can provide evidence-based medicine recommendations for MST treatment of post-stroke rehabilitation Patien.

Research limitations: First of all, in the process of MST treatment, the choice of treatment, the choice of treatment site, time and frequency may be heterogeneous. Second, this study has set strict inclusion criteria, and the inclusion of high-quality literature may have less impact. The reliability of systematic review depends to a large extent on comprehensiveness and methodological quality. Third, the included studies do not limit language types, and there are certain language biases.

## Author contributions

**Conceptualization:** Jie Ding, YuanYi Xiao, Jinyu Hu.

**Data curation**: Jie Ding, Jinyu Hu, YuanYi Xiao.

**Formal analysis:** Jie Ding, YuanYi Xiao, Fuqiang Yuan, Jinyu Hu.

**Funding acquisition:** Jie Ding.

**Investigation**: Yuanyi Xiao, Jinyu Hu, Jie Ding.

**Methodology**: Yuanyi Xiao, Jinyu Hu, Fuqiang Yuan.

**Project administration**: Jie Ding, Jinyu Hu, Fuqiang Yuan.

**Resources:** Jinyu Hu.

**Software**: Yuanyi Xiao, Jinyu Hu.

**Supervision:** Zhaona Luo, Jinyu Hu, Jie Ding.

**Validation:** Zhaona Luo, Jinyu Hu, Jie Ding.

**Visualization:** Zhaona Luo, Jinyu Hu.

**Writing – original draft**: Jie Ding, Jinyu Hu.

**Writing – review & editing**: Yuanyi Xiao, Jinyu Hu.
